# THE IMPACT OF DISCRIMINATORY POLICIES AND PRACTICES ACROSS THE AGING CONTINUUM

**DOI:** 10.1093/geroni/igz038.1637

**Published:** 2019-11-08

**Authors:** Brian W Lindberg

**Affiliations:** The Gerontological Society of America, Washington, District of Columbia, United States

## Abstract

Aging crosses all domestic and international borders. It’s an issue that affects everyone regardless of religion, race, nationality, ethnicity, sexual orientation, or gender identity. The main purpose of The Gerontological Society of America (GSA) is “to advance the scientific and scholarly study of aging and to promote human welfare by the encouragement of gerontology in all its areas.” Yet in 2019, policies remain in effect that impact individuals in a discriminatory manner. The program will highlight research in several areas that demonstrate the effects of these discriminatory practices.

